# Effects of Terminal Motif on the Self-Assembly of Dexamethasone Derivatives

**DOI:** 10.3389/fchem.2020.00009

**Published:** 2020-02-20

**Authors:** Hui Liu, Ailing Yu, Mali Dai, Dan Lin, Deqing Lin, Xu Xu, Xingyi Li, Yuqin Wang

**Affiliations:** School of Ophthalmology & Optometry and Eye Hospital, Institute of Biomedical Engineering, Wenzhou Medical University, Wenzhou, China

**Keywords:** dexamethasone, self-assembly, terminal motif, drug delivery, hydrogel

## Abstract

Tailoring the terminal motif of molecules including drugs might significantly affect their self-assembly tendency in aqueous solution, thus providing a rational strategy to modulate its macroscopic characteristics of supramolecular assembly. A model drug of dexamethasone (Dex) was esterified by different fatty acids [succinic acid (SA), glutaric acid (GA), and adipic acid (AA)] and aromatic acid [phthalic acid (PA)] to generate a series of Dex derivatives. Aqueous solution of Dex-SA, Dex-GA, and Dex-AA turned into hydrogel spontaneously after a period time of incubation (24, 48, and 72 h, respectively) via the auto-hydrolytic strategy, while aqueous solution of Dex-PA did not result in hydrogelation during 3 days of incubation. Aqueous solutions of Dex-SA, Dex-GA, and Dex-AA underwent apparent hydrolysis (10.73 ± 0.64%, 15.17 ± 2.24%, and 17.29 ± 1.39%, respectively), while Dex-PA exhibited a minimal hydrolysis (<1%) in a period of 28 days study, as indicated by *in vitro* hydrolytic test. Morphological observation showed that the hydrogel formed by Dex-SA was composed of uniform nanofibers, while hydrogels formed by Dex-GA, and Dex-AA were derived from irregular particles. The mechanical strength of hydrogel formed by Dex-SA was much bigger than that of hydrogels formed by Dex-GA and Dex-AA, as indicated by rheological test. Moreover, the acylation of Dex did not compromise its potent anti-inflammatory activity in a lipopolysaccharide (LPS)-activated RAW 264.7 macrophage.

## Introduction

Self-assembly of nanomaterials from molecular building block is a promising approach to construct new functional materials for various biomedical applications (e.g., drug delivery, tissue engineering; Blanco et al., 2015; Kalepu and Nekkanti, 2015; Adawiyah et al., 2016; Vader et al., 2016; Zylberberg and Matosevic, 2016; Wu et al., 2017b). Self-assembly is primarily dominated by various non-covalent interactions such as electrostatic, hydrophobic, hydrogen bond, and van der Walls interactions (Zhou and Li, 2017; Yang et al., 2018). The biggest challenge in molecular self-assembly is to precisely control the self-assembly process (nucleation and growth of nanostructure; Zhao et al., 2009; Vemula et al., 2013). In the past several decades, a number of strategies including enzyme, pH condition, light, and so on have been proposed to achieve a spatial and temporal controlled self-assembly of nanomaterials (e.g., peptide, lipid; Vemula et al., 2009, 2013; Mao et al., 2012; Li et al., 2013; Lin and Cui, 2015; Ma et al., 2016; Wang et al., 2017b; Wu et al., 2018). Among these strategies, approaches involving hydrolytic or enzymatic reactions have gained considerable attention, because they offered great opportunities to elaborately tailor the characteristics of assemblies.

One emerging class of self-assembly system is steroidal agents (e.g., bile salts, sodium deoxycholate) that form numerous supramolecular assemblies (e.g., fiber, tube, particle) and have been reported for a long time (Terech et al., 2002, 2009; Svobodová et al., 2012; Zhang et al., 2017). Similarly, steroidal drugs (e.g., dexamethasone, triamcinolone acetonide) have been widely used to construct supramolecular hydrogel owing to their excellent self-assembly capacity (Wu et al., 2017a; Xiong et al., 2018; Zhou et al., 2018). For instance, Samuel I. Stupp and co-workers reported a dexamethasone-peptide amphiphile that was able to self-assemble into nanofibers via ionic cross-linking strategy, acting as a novel drug delivery system to provide prolonged and localized drug delivery at the site of injection (Webber et al., 2012). More recently, we occasionally found that the succinated dexamethasone (Dex-SA) underwent auto-hydrolysis in phosphate buffered saline (PBS, pH 7.4) to generate a transparent hydrogel (Zhang et al., 2018). Notably, gelation time and mechanical strength of Dex-SA supramolecular hydrogel can be readily modulated by the pH condition of medium and incubation temperature, as well as the hydrogelator concentration. However, since these parameters dramatically influence the macroscopic properties of hydrogels, the effect of terminal motif on the self-assembly behaviors of dexamethasone has not yet been reported. In this study, we investigated the effects of terminal motif on the self-assembly of dexamethasone derivatives, thus providing the basis to precisely modulate macroscopic characteristics in the supramolecular assemblies.

## Materials and Methods

### Materials

Dex and succinic anhydride were purchased from J&K Scientific Ltd (Beijing, China). Glutaric anhydride was purchased from Beijing Bailingwei Technology Co., Ltd. (Beijing, China). Adpic anhydride was purchased from Nanjing Kangmanlin Biology Technology Co., Ltd. (Nanjing, China). Phthalic anhydride was purchased from Adamas-beta Co., Ltd. (Shanghai, China). Mouse TNF-α DuoSet ELISA (DY410-05) and mouse IL-6 DuoSet ELISA (DY406-05) were obtained from R&D SYSTEMS® (Minneapolis, MN, USA). All other used agents were of analytical grade.

### Synthesis and Characterization of Dex Derivatives

As reported previously (Zhang et al., 2018), Dex-SA, Dex-GA, Dex-AA, and Dex-PA derivatives were synthesized via an esterification reaction at room temperature. Briefly, Dex, and anhydrides with molar ratio at 1:3 were co-dissolved into pyridine for reaction at room temperature overnight. After that, the pyridine was evaporated using a rotary evaporator, and the resulting residues were recrystallized in petroleum ether to afford a series of Dex derivatives. Finally, the obtained products were further confirmed by LC-MS and ^1^H-NMR (Figures S1–S8).

### Self-Assembly of Dex Derivatives

An indicated amount of Dex derivatives (Dex-SA, Dex-GA, Dex-AA, and Dex-PA) were suspended in PBS (pH 7.4), followed by the addition of a Na_2_CO_3_ solution (1 equiv to Dex derivatives) to afford a transparent solution at a final concentration of 2 wt%. Sol–gel transition was monitored by an inverted test tube method as reported previously (Joo et al., 2007).

### *In vitro* Hydrolytic Study

The hydrolytic ratio of Dex-SA, Dex-GA, and Dex-AA supramolecular hydrogels and Dex-PA aqueous solution as a function with time was measured by the quantification of the hydrolytic product Dex using HPLC assay. Briefly, 1 ml of Dex-SA, Dex-GA, Dex-AA, and Dex-PA aqueous solution was stored at room temperature for a period of 28 days of study. At predetermined time points, 0.01 ml aliquot of samples were collected and the hydrolytic product Dex was quantified by high-performance liquid chromatography (HPLC, Agilent 1290) with a reversed-phase C18 column (ZORBAX Eclipse XDB-C18, 150 × 4.6 mm i.d., 5 μm, Agilent). The mobile phase for the detection of Dex-SA, Dex-GA, and Dex-AA was composed of methanol and 0.1% acetic acid (60/40; v/v), while the mobile phase for the measurement of Dex-PA was composed of methanol and 0.1% acetic acid (70/30; v/v) at a flow rate of 1 ml/min. Aliquots of 20 μl of tested samples were injected for HPLC analysis. Detection was performed by a diode array detector (DAD) at 220 nm. The hydrolytic ratio of substrate was calculated using the following equation: Hydrolytic ratio (%) = The amount of Dex at the indicated time point/Total amount of substrate × 100.

### Morphology Observation

The morphology of Dex-SA, Dex-GA, and Dex-AA supramolecular hydrogel and Dex-PA aqueous solution was observed by transition electron microscopy (TEM). Samples were pipetted onto a copper grid and stained with a 0.5 wt% phosphotungstic acid solution for TEM observation.

### Rheology Test

The rheological properties of Dex-SA, Dex-GA, and Dex-AA supramolecular hydrogels were measured using a TA-AR 2000 rheometer (New Castle, DE, USA). A 40-mm cone plate was used for the experiment. A 0.4 ml hydrogel sample was loaded and a frequency sweep from 0.1 to 100 rad/s was performed.

### *In vitro* Anti-inflammatory Efficacy

To assess the *in vitro* anti-inflammatory efficacy of Dex derivatives (Dex-SA, Dex-GA, Dex-AA, and Dex-PA), we measured the pro-inflammatory cytokine levels in the culture medium using LPS-activated RAW264.7 macrophages. Briefly, RAW264.7 macrophages were seeded at a density of 0.7 × 10^4^ cells per well in a 24-well plate and pre-treated by 10 μM Dex, Dex-SA, Dex-GA, Dex-AA, and Dex-PA aqueous solution for 2 h, followed by the challenge with 1000 ng/ml lipopolysaccharide (LPS) for 24 h. Thereafter, the supernatant from each well was collected, and the pro-inflammatory factors including tumor necrosis factor-α (TNF-α) and interleukin-6 (IL-6) levels were measured using an ELISA kit (Neobioscience, Shenzhen, China). The level of nitrite (NO) was detected by the Griess assay as reported previously (Zhou et al., 2018). Cells without any manipulation were used as the negative control.

### Statistical Analysis

The values of all experiments were expressed as the mean ± SD and statistically analyzed by Origin 8.5. *In vitro* anti-inflammatory efficacy data were analyzed by one-way analysis of variance (ANOVA) with Tukey's multiple comparisons test using GraphPad Prism 7.00. Statistical significance was considered at a probability level of *P* < 0.05.

## Results and Discussion

### Self-Assembly of Dex Derivatives

**Figure 1:**
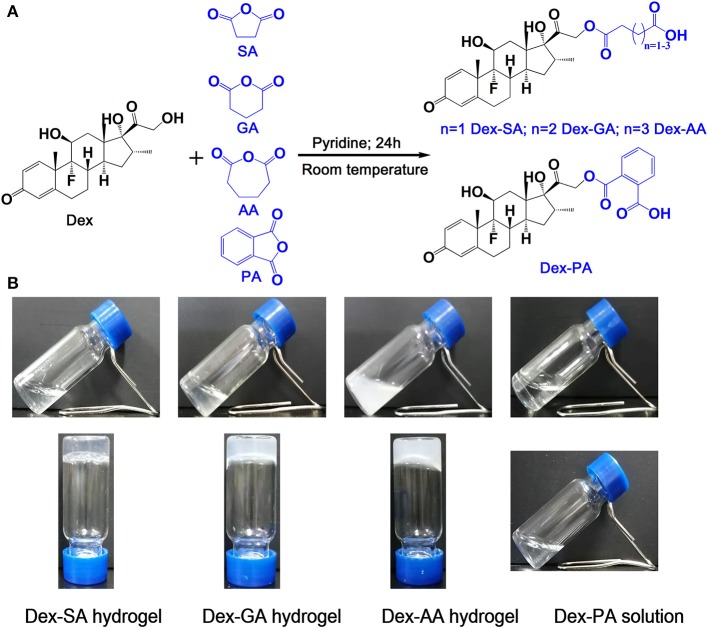
**(A)** Synthetic scheme of Dex-SA, Dex-GA, Dex-AA, and Dex-PA. **(B)** Appearance of Dex-SA, Dex-GA, and Dex-AA hydrogels and Dex-PA aqueous solution; Dex-SA, Dex-GA, and Dex-AA hydrogels formed at 24, 48, and 72 h with a final concentration at 2 wt%.

Pilot studies have illustrated that the terminal motif of molecules (e.g., drugs, peptide) exhibited a strong influence over the self-assembling capacity of the same self-assembling core (Zhang et al., 2010; Ryan et al., 2011; Wang et al., 2017a). We therefore rationally designed and synthesized four precursors of Dex (Figure 1A; Dex-SA, Dex-GA, Dex-AA, and Dex-PA, respectively). We speculated that the acyl and alkyl chain lengths of the four precursors might dramatically influence the self-assembly tendency of Dex, thus resulting in precisely control macroscopic properties of supramolecular assemblies. We thereafter dissolved the compounds into PBS (pH 7.4) at a final concentration of 2 wt% (20 mg/ml). It is clearly observed that the Dex-SA, Dex-GA, and Dex-PA resulted into a transparent solution in PBS, while the Dex-AA exhibited a translucent solution (Figure 1B). Aqueous solutions of Dex-SA, Dex-GA, and Dex-AA turned into hydrogels upon incubation at ambient temperature for 24, 48, and 72 h, respectively. The resulting three hydrogels exhibited different appearances. Hydrogel formed by Dex-SA is transparent, and hydrogels formed by Dex-GA and Dex-AA are turbid (Figure 1B). Notably, the hydrogels formed by Dex-GA and Dex-AA occurred the precipitation after 7 days of incubation, while the hydrogel formed by Dex-SA was very stable even up to 7 days storage (Data not shown). Interestingly, there were no obvious changes in aqueous solution of Dex-PA during 3 days of incubation at ambient temperature. All these results suggested that the acyl and alkyl chain lengths had a significant effect on the self-assembly behavior of Dex conjugates.

### Hydrolytic Study

**Figure 2:**
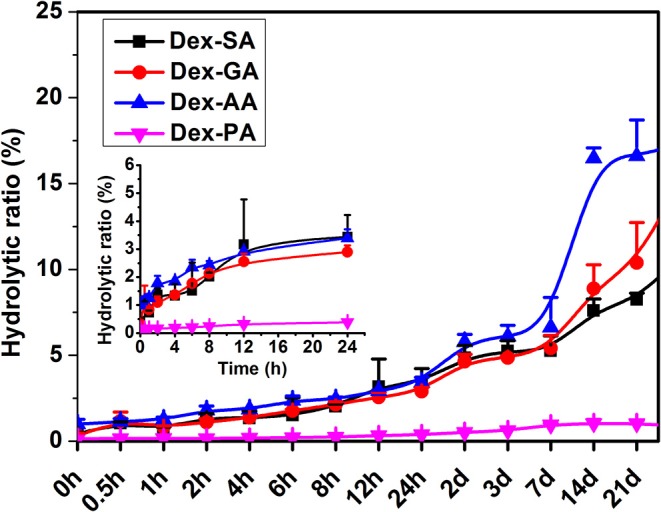
Hydrolytic ratio of Dex-SA, Dex-GA, Dex-AA, and Dex-PA after incubation in phosphate buffered saline (PBS; pH 7.4) at room temperature.

The hydrolytic ratio of various Dex conjugates (Dex-SA, Dex-GA, Dex-AA, and Dex-PA) was monitored by measuring the hydrolytic product Dex by HPLC assay. As shown in Figure 2, it is clearly observed that Dex-SA, Dex-GA, and Dex-AA underwent similar hydrolytic behavior with about 3% hydrolysis of total substrate after 24 h incubation in PBS (pH 7.4). It seems to suggest that the hydrolytic rate of Dex-SA, Dex-GA, and Dex-AA was very slow in PBS solution and alkyl chain lengths in the range of 4–6 had no effect on the hydrolytic rate of ester bond. By extending incubation to 28 days, the hydrolytic rate of Dex-SA, Dex-GA, and Dex-AA accelerated significantly and the hydrolytic ratio of Dex-SA, Dex-GA, and Dex-AA achieved 10.73 ± 0.64%, 15.17 ± 2.24%, and 17.29 ± 1.39%, respectively. Interestingly, Dex-PA resulted in a distinct reduction of the hydrolytic rate in PBS, and the hydrolytic ratio of Dex-PA was <0.5% after 24 h incubation. Even after 28 days of incubation, the hydrolytic ratio of Dex-PA was still <1%, indicating the relatively stable Dex-PA in PBS solution. This result was in accordance with the previous report of aromatic esters that are relatively stable over the fatty acid esters in most cases owing to the steric hindrance of the aromatic group to increase the activation energy of the hydrolysis (Khuwijitjaru et al., 2004).

### Morphological Observation

**Figure 3:**
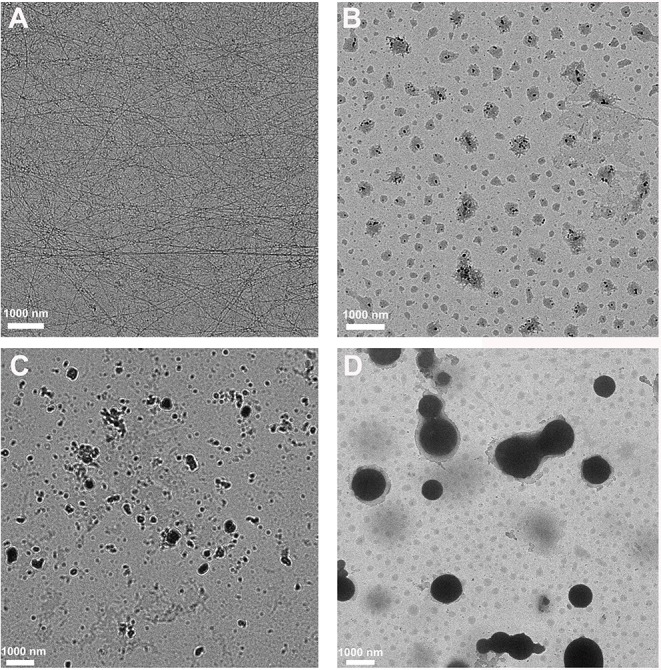
TEM images of **(A)** Dex-SA hydrogel, **(B)** Dex-GA hydrogel, **(C)** Dex-AA hydrogel, and **(D)** Dex-PA aqueous solution.

We then adopted transmission electron microscopy (TEM) to visualize the morphology of nanostructures in the resulting hydrogels or solution. As shown in Figure 3A, Dex-SA hydrogel displayed the dense and three-dimensional networks of nanofibers. Unlike the Dex-SA hydrogel, Dex-GA, and Dex-AA hydrogels were composed of some irregular particles (Figures 3B,C), which tends to aggregate, resulting in the precipitation after 7 days of incubation. It is not surprising that Dex-PA solution was composed of spherical particles ranging 600–1,200 nm in size (Figure 3D). These results indicated that the acyl and alkyl chain lengths exert a profound influence on self-assembly propensity and morphology.

### Rheology Test

**Figure 4:**
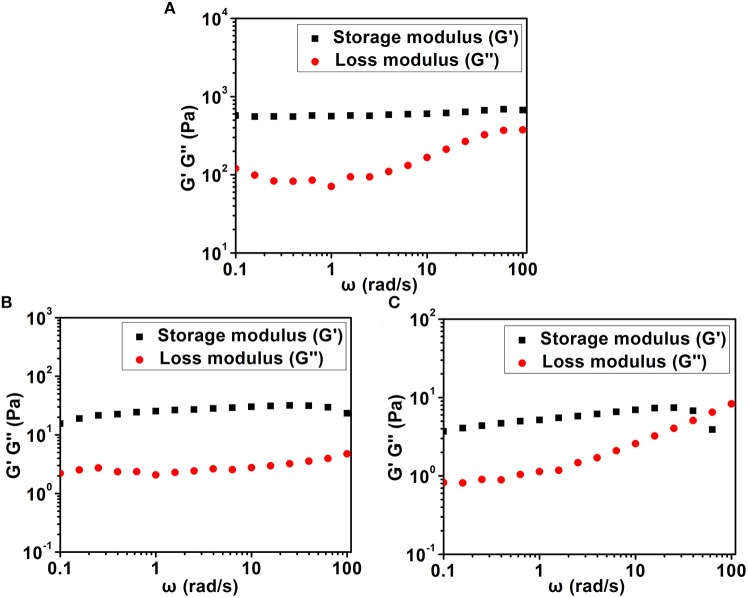
Dynamic frequency sweep of **(A)** Dex-SA hydrogel, **(B)** Dex-GA hydrogel, and **(C)** Dex-AA hydrogel at a strain of 1%.

We then used a rheometer to characterize the mechanical properties of various hydrogels. As shown in Figure 4, all hydrogels exhibited weak frequency dependence in the range of 0.1–100 rad/s, suggesting the relative elasticity of the hydrogels. More importantly, the storage modulus (*G'*) value of hydrogel formed by Dex-SA was much larger than that of hydrogel formed by Dex-GA and Dex-AA. For instance, the *G'* value was about 560, 25, and 5 Pa at a frequency of 1 rad/s for hydrogels formed by Dex-SA, Dex-GA, and Dex-AA, respectively. The relatively higher mechanical strength of hydrogel formed by Dex-SA might be ascribed to the formation of nanofibers to support its three-dimensional structure (Figure 3A). These results strongly indicated that the mechanical strength of hydrogels were also greatly influenced by the terminal motif of Dex.

### *In vitro* Anti-inflammatory Test

**Figure 5:**
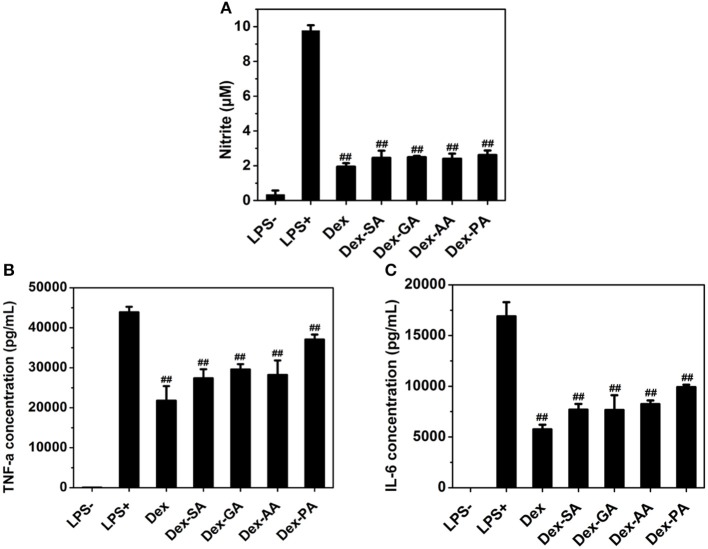
**(A)** NO **(B)** TNF-α, and **(C)** IL-6 expression in cell culture medium after treatment by 10 μM Dex, Dex-SA, Dex-GA, Dex-AA, and Dex-PA in LPS-actived RAW 264.7 macrophage; ^**##**^*p* < 0.05 vs. LPS-activated group.

Since macrophage played a very important role in the inflammatory response via the secretion of a variety of factors (Perry et al., 1993; Mantovani et al., 2005; Cohen and Mosser, 2013), such as nitric oxide (NO) and pro-inflammatory cytokines (e.g., IL-1β, TNF-α, IL-6) after the challenge with an activating stimulus (e.g., lipopolysaccharide), we have investigated the anti-inflammatory activity of various Dex conjugates in an LPS-activated RAW 264.7 macrophage. As presented in Figure 5, it was clearly observed that the untreated RAW 264.7 macrophage produced low levels of nitrite (NO), IL-6, and TNF-α in cell culture medium, while the activated RAW 264.7 macrophage by 1000 ng/ml LPS resulted in the high release of NO, IL-6, and TNF-α to the culture medium. Pretreatment with various Dex conjugates (Dex, Dex-SA, Dex-GA, Dex-AA, and Dex-PA) gave rise to the significant reduction of the NO, IL-6, and TNF-α production to cell culture medium (^**##**^*p* < 0.05 vs. LPS-activated group), suggesting that the acylation of Dex did not compromise its potent anti-inflammatory activity.

## Conclusion

The terminal motif of Dex significantly affects the self-assembly behavior of Dex in aqueous solution. Aqueous solution of Dex-SA, Dex-GA, and Dex-AA turned into hydrogel within 72 h of incubation, and Dex-PA solution kept the clear solution within 7 days of incubation. Dex-SA, Dex-GA, and Dex-AA exhibited a progressive hydrolysis in PBS solution for a period of 28 days study, and Dex-PA did not result in the apparent hydrolysis within 28 days of incubation. Hydrogel formed by Dex-SA was composed of dense and uniform nanofibers, while hydrogels formed by Dex-GA and Dex-AA were made of irregular particles. More importantly, the acylation of Dex did not compromise its potent anti-inflammatory activity in an LPS-activated RAW 264.7 macrophage. Based on these results, we provided the basis to rationally tailor the terminal motif of drugs and thus precisely modulate the self-assembly behavior of Dex conjugates and control the macroscopic characteristics of its supramolecular assemblies without compromising its pharmacological activities.

## Data Availability Statement

All datasets generated for this study are included in the article/Supplementary Material.

## Author Contributions

XL and YW contributed to the conception and design of the study. HL performed the study and organized the database. XX performed the statistical analysis. HL and AY wrote the first draft of the manuscript. MD, DaL, and DeL wrote sections of the manuscript. All authors contributed to manuscript revision, and read and approved the submitted version.

### Conflict of Interest

The authors declare that the research was conducted in the absence of any commercial or financial relationships that could be construed as a potential conflict of interest.
