# Supplementary material for: Effects of Terminal Motif on the Self-Assembly of Dexamethasone Derivatives
Source: Front Chem. 2020 Feb 20;8:9. doi: 10.3389/fchem.2020.00009 (PMC7044695; doi:10.3389/fchem.2020.00009)
Supplement: Supplementary file 1 [file Data_Sheet_1.pdf]

# *Supplementary Material*

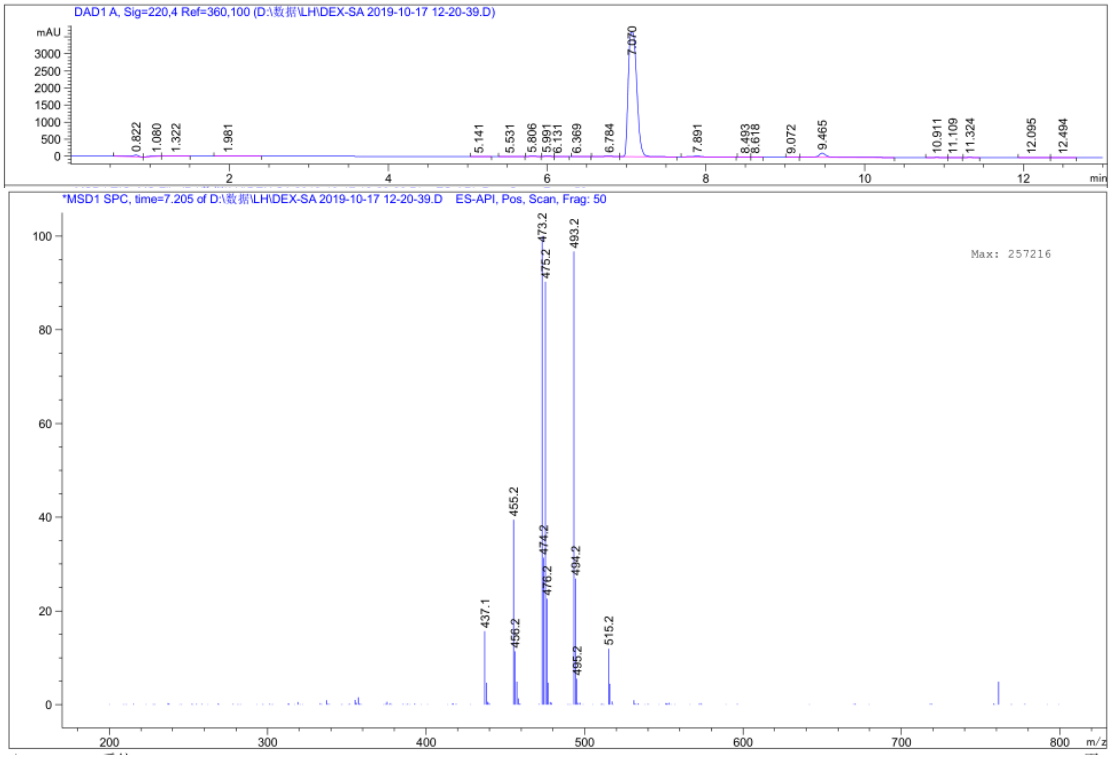


**Fig.S1** LC-MS spectrum of Dex-SA conjugate

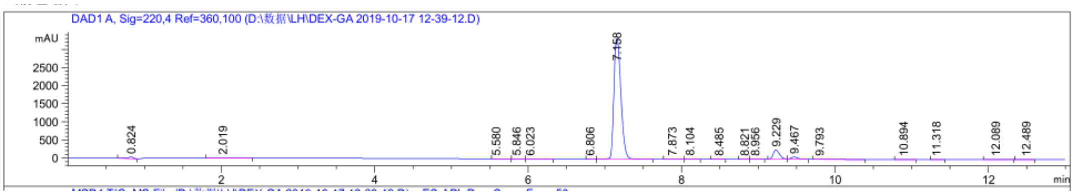


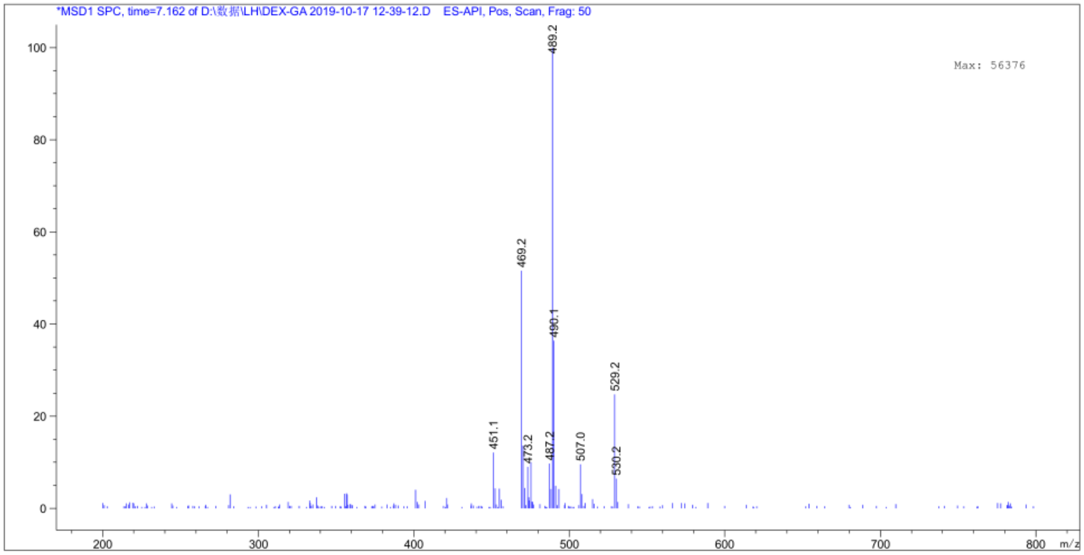


**Fig.S2** LC-MS spectrum of Dex-GA conjugate

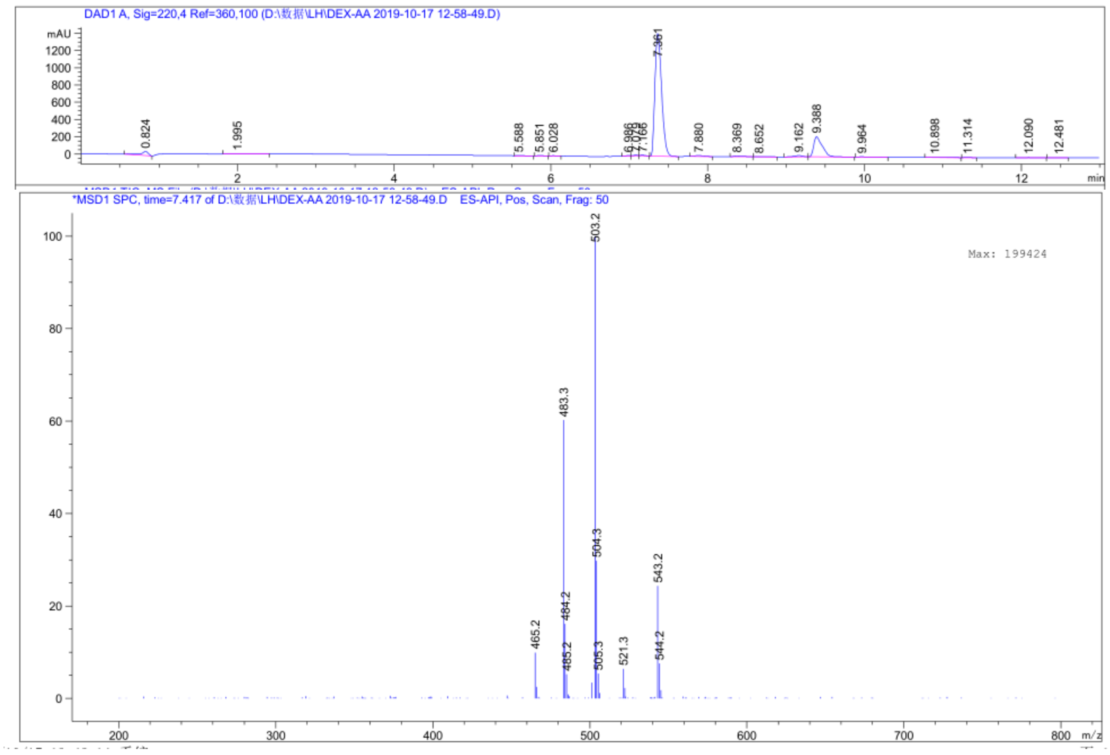


**Fig.S3** LC-MS spectrum of Dex-AA conjugate

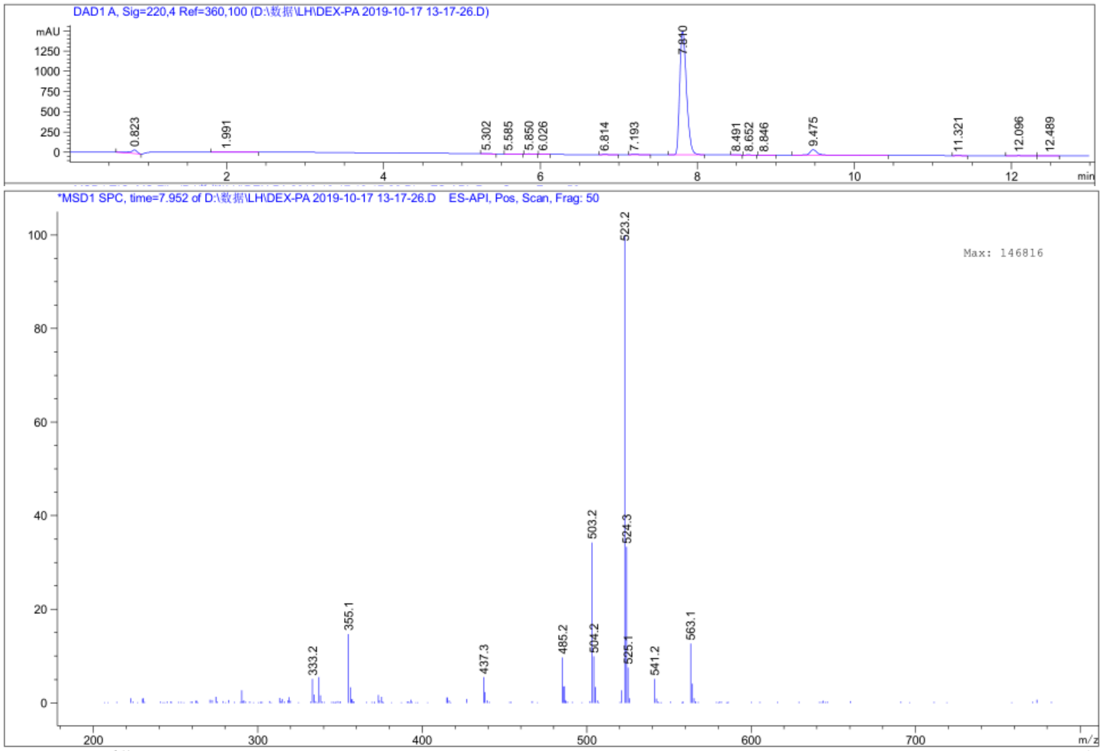


**Fig.S4** LC-MS spectrum of Dex-PA conjugate

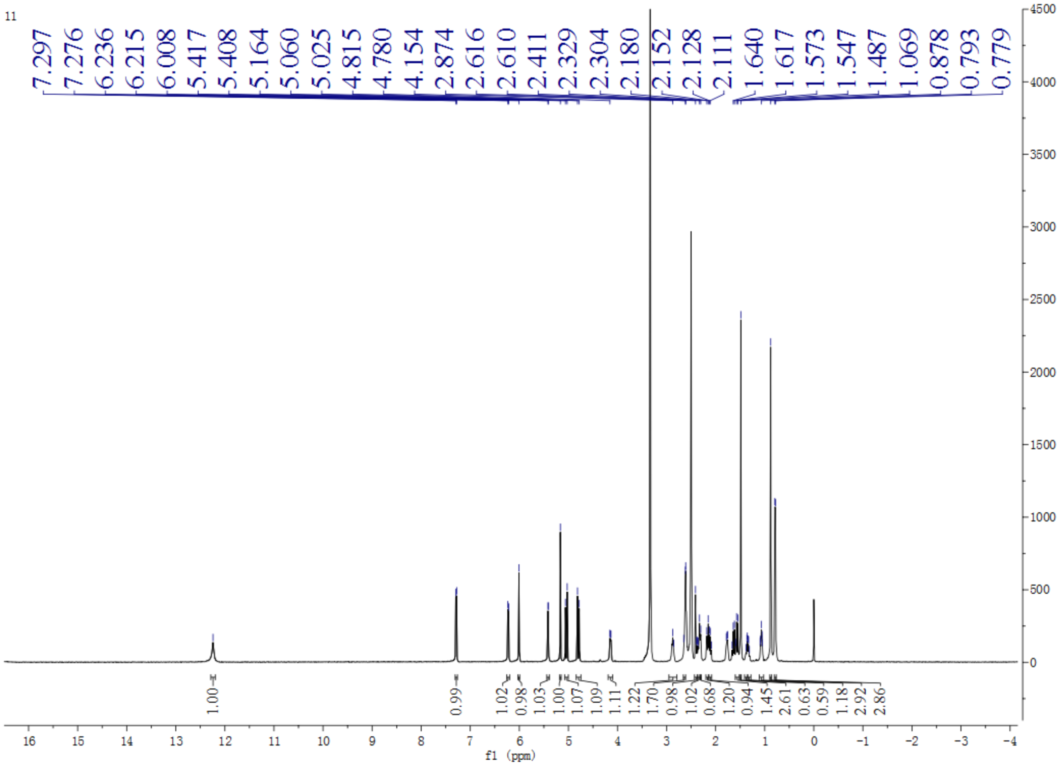

**Fig.S5** $^{1}$H-NMR spectrum of Dex-SA conjugate

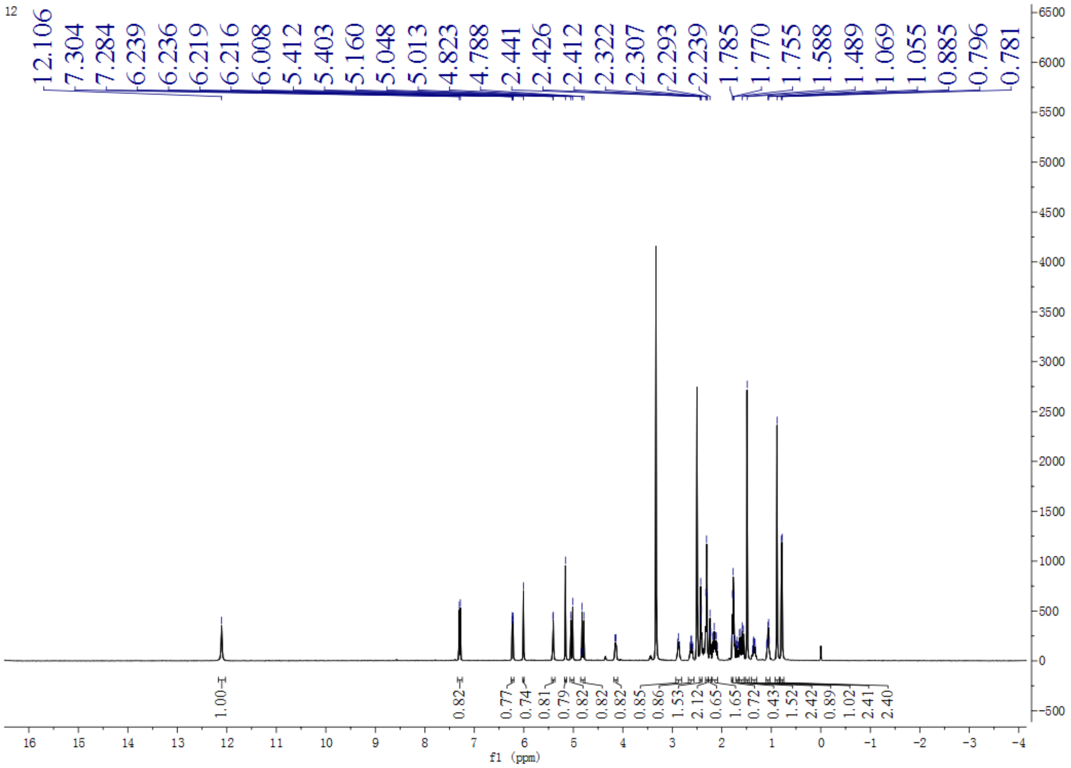

**Fig.S6** $^{1}$H-NMR spectrum of Dex-GA conjugate

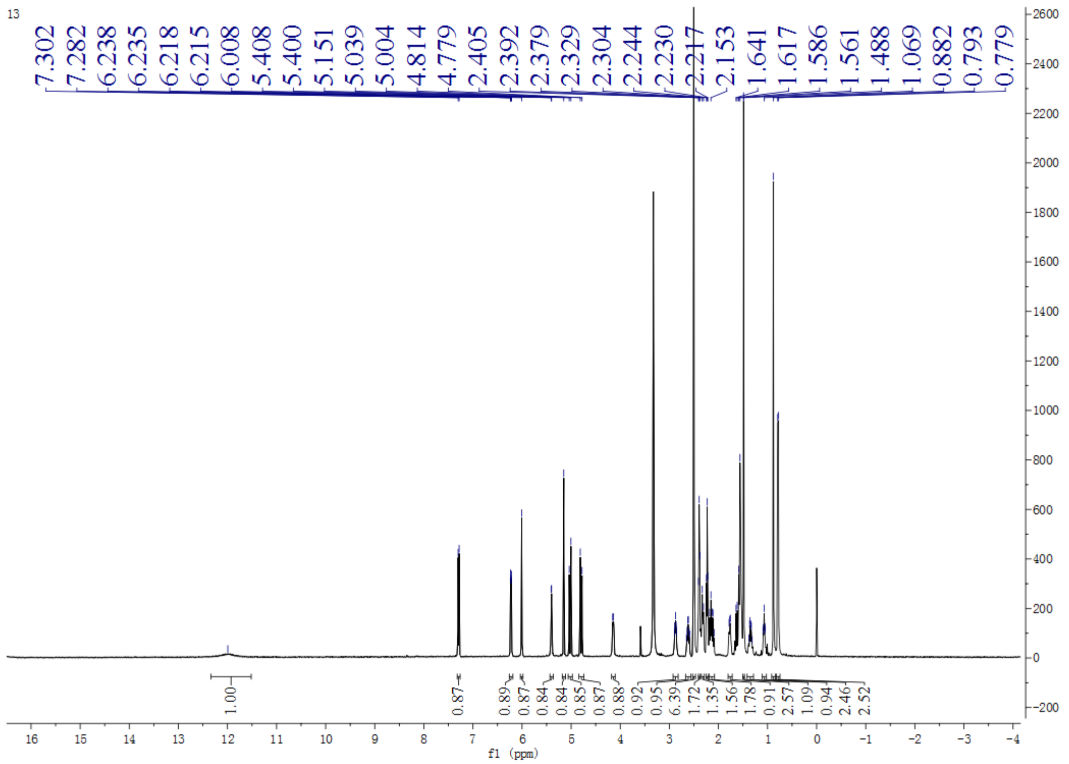


**Fig.S7** $^{1}$H-NMR spectrum of Dex-AA conjugate

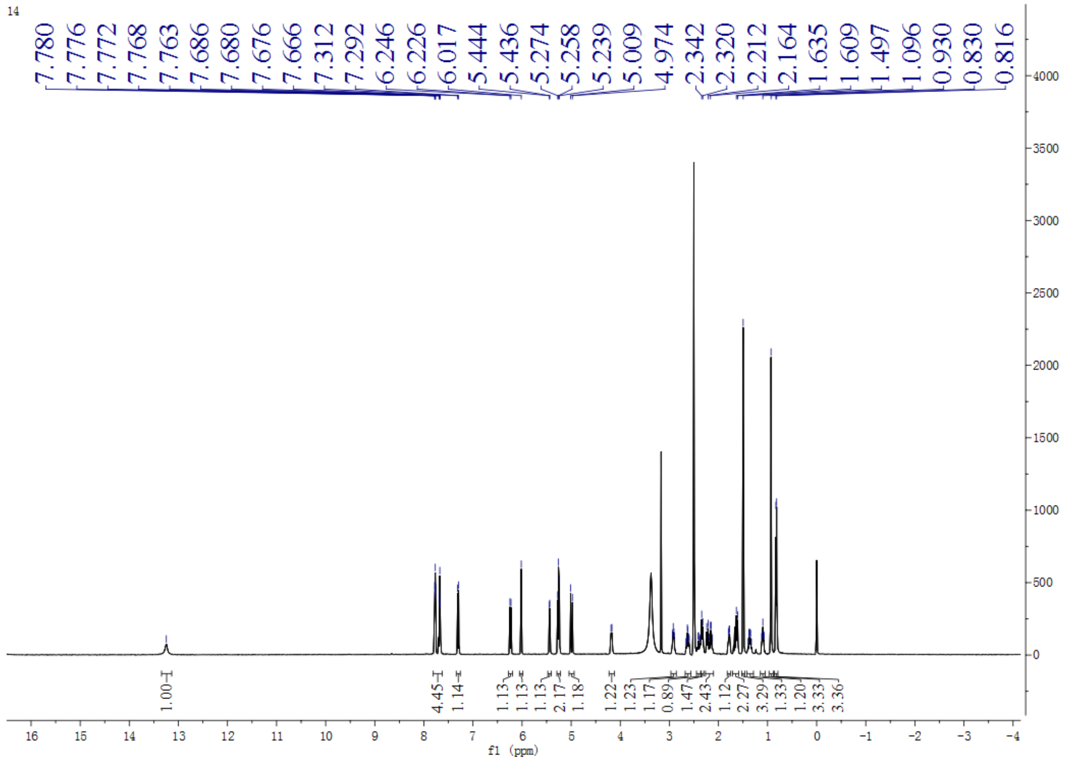


**Fig.S8** $^{1}$H-NMR spectrum of Dex-PA conjugate
